# Use of Statins as Lipid Lowering Agent in Hypercholesterolemia in a Tertiary Care Hospital: A Descriptive Cross-sectional Study

**DOI:** 10.31729/jnma.5444

**Published:** 2020-12-31

**Authors:** Ashish Kumar Bhattarai, Anna Acharya, Prabin Kumar Karki

**Affiliations:** 1 Department of Pharmacology, Kathmandu Medical College, Duwakot, Bhaktapur, Nepal; 2 Department of Physiology, Kathmandu Medical College, Duwakot, Bhaktapur, Nepal

**Keywords:** *dyslipidemia*, *hydroxymethylglutaryl-Co A reductase*, *statins*

## Abstract

**Introduction:**

Lipids contribute to atherosclerosis and obesity that can lead to different cardiovascular diseases. Statins are hydroxymethylglutaryl reductase inhibitors that effectively lower the cholesterol level. It is widely prescribed in the treatment of hypercholesterolemia. Thus, it optimizes the lipoprotein profile. Selection of a particular drug by the practitioner should be primarily based on clinical outcome. This study was conducted to find type of statins which are most preferred by the doctors for treating dyslipidemia and preferred the fixed-dose in a tertiary care hospital.

**Methods:**

This was a descriptive cross-sectional study conducted among the practicing doctors of Kathmandu Medical College from July to August 2020. Ethical approval was taken from the Institutional Review Committee of the college (Ref: 207202006). Convenient sampling was done. A semi-structured questionnaire was used with consent. The data were analyzed with Social Statistical Package for the Social Sciences version 20.

**Results:**

Statins, with the score 4.25 was accounted for the most preferred for the treatment of dyslipidemia. Among different statins, atorvastatin with a score of 4.48 was most popular followed by rosuvastatin 2.9 score and simvastatin 2.1 score.

**Conclusions:**

Statins was the most preferred agents for the treatment of dyslipidemia. Although different types of statins ought to have similar efficacy treating dyslipidemia, atorvastatin was found to be popular and most commonly prescribed one. The most common side effect reported with statins was myopathy.

## INTRODUCTION

Lipids in the body contribute to atherosclerosis and obesity. High lipids in the body may cause a high cholesterol level. These commonly cause coronary and cerebrovascular diseases, two major morbidities worldwide. Also, the adipokines released by these adipose tissues in the body induces insulin resistance, hypercoagulability, and systemic inflammation.^1^ HMG-CoA (3-hydroxy-3methylglutaryl-coenzyme A) reductase required for the cholesterol biosynthesis. To reduce the cholesterol level, the drug-like statins which are HMG-CoA reductase inhibitors are widely prescribed in the treatment of hypercholesterolemia.^2–3^

Even if the clinical effects of statins are similar potentiating therapeutic equivalence, they can differ in pharmacokinetic properties and interaction potentials.^4^ Selection of a particular drug by the practitioner should be primarily based on clinical outcome, keeping in account the costs and pharmacokinetic profile including the interaction potential and existence of other co-morbid conditions.

The objective of this study was to know the preference of the type of statins by the doctors for treating dyslipidemia including preferred combinations in a tertiary care center.

## METHODS

This was a descriptive cross-sectional study conducted in the Kathmandu Medical College. It was conducted among the doctors practicing at Kathmandu Medical College from July to August 2020. This study was approved by the Institutional Review Committee of the Kathmandu Medical College (Ref: 207202006) . Doctors including the medical officers, residents, consultants who are treating dyslipidemia, and willing to participate in this study where included. Those doctors who have not given consent, in the off duty and the doctors involved in the pretesting were excluded from the study. The sample size was calculated by using a formula,

$$\begin{array}{ll} \text{n} & \text{= }{\text{Z}}^{\text{2}}\times \text{p}\times \text{q}/{\text{e}}^{\text{2}}\\ & \text{= }{\left(1.96\right)}^{\text{2}}\times (0.5)\times (1-0.5)/{(\text{0.15)}}^{\text{2}}\\ & \text{= }43 \end{array}$$

where,

n = sample size
Z = 1.96 at 95% Confidence Interval
p = prevalence, 50%
q = 1-p
e = margin of error, 15%

The calculated sample size is 43. Taking the non-response rate of 5%, the sample size is 45. Convenient sampling was done. A semi-structured, undisguised questionnaire consisting of both open and closed- ended items were used. The questionnaire was inspired by the published article^5^ and the questionnaire was finalized by the faculty members of the Department of Pharmacology for content validation. It was pre-tested among the small group of seven doctors to look for the understanding and acceptance for the face validation. These seven doctors were not included in the study. Based on the response of the pretesting, after minor modification, the final questionnaire was prepared and distributed.

A ten-item semi-structured questionnaire comprising two sections was used to achieve the study objective. First section: comprised the three items about the demographic information of the prescriber. Section two: this section was to evaluate the extent and pattern of the use of the Statins. It included the number of patients treated by the doctor in a week and a more prevalent sex group according to their experience. The third question included the preferred group of drugs for dyslipidemia. The preference was scored from 5 to 1; 5 being most preferred and 1 being least preferred among five drug names given by the doctors. The average value obtained from the scoring was utilized to rank the drugs. Preference was evaluated with their different basic like group, generic name, and fixed- dose combinations of the medication.

After the informed written consent taken from the participants, the questionnaire was distributed to all the doctors including the medical officers, residents, consultants who are treating dyslipidemia, and willing to participate in this study. The participant's identity was not disclosed.

Data was collected, compiled, and analyzed by using the Statistical Package of Social Science (SPSS) version 20. Descriptive data were expressed as a percentage, frequency. The results were tabulated where necessary.

## RESULTS

The total number of participating doctors was 45. Of them, 20 (44.4%) were consultants in the Department of Medicine and Emergency and the remaining 25 (55.5%) medical officers working in different departments of Kathmandu Medical College. Of the participants, 30 (66.6%) were male candidates, while 15 (33.3%) were female candidates.

Most doctors 20 (44.4%) prescribed less than 5 patients in a week followed by 14 (31.1%) doctors who prescribed 6 to 10 patients in a week and 5 (11.1%) doctors prescribed more than 20 cases in a week (Table 1).

**Table 1:** The average number of patients of dyslipidemia treated by the doctors in a week (n = 45).

Patients prescribed by the doctor	n (%)
1-5	20 (44.4)
6-10	14 (31.1)
11-15	4 (8.8)
16-20	2 (4.4)
>20	5 (11.1)

The 32 (71.1%) doctors' found male patients more prevalent for dyslipidemia while 13 (28.9%) doctors thought females are more prone to have dyslipidemia based on their practice.

When a different group of drugs was listed in the questionnaire form, the participants ranked their drug of choice giving 5 points to the most preferred and 1 to the least preferred drug. The ranking was done after the average was calculated for each drug. Statins were found to be the most preferred group of drugs with an average score of 4.25 followed by fibrates 3.18 score, and ezetimibe 2.1 score (Table 2).

**Table 2:** A preferred group of the prescribed drug and their ranking for the treatment of dyslipidemia.

Drug	Average score (Out of 5)	Ranking
Statins	4.25	1
Fibrates	3.18	2
Ezetimibe	2.1	3
Nicotinic acid	1.7	4
Bile acid binding agent	1.6	5

The preferred statins among different available in the market by participants ranked by the participants. Considering the average score, atorvastatin was found to be the most preferred type of drug with average score 4.48 followed by rosuvastatin 2.9 score, and simvastatin 2.1 (Table 3).

**Table 3:** Preferred Statin among different available in the market and their ranking.

Drug	Average Score (Out of 5)	Ranking
Atorvastatin	4.48	1
Rosuvastatin	2.9	2
Simvastatin	2.1	3

**Figure 1:**
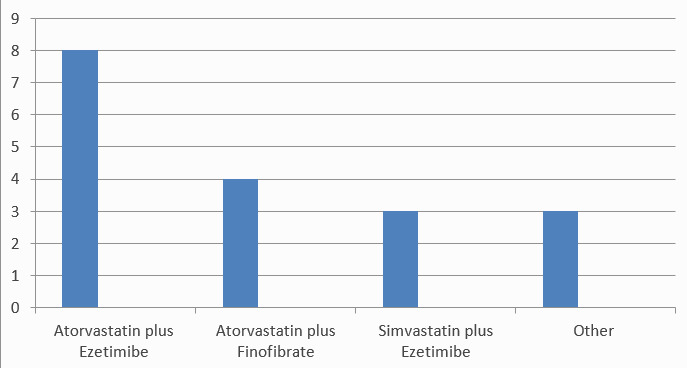
Status of preference for different combinations of statins.

Among 45 participants, 27 (60%) did not prefer to give any combination of drugs. Maximum participants who preferred the combination drugs choose atorvastatin and ezetimibe combination 8 (17.7%), followed by atorvastatin and fenofibrate 4 (8.88%) and simvastatin and ezetimibe 3 (6.66%). The other option included atorvastatin and nicotinic acid, atorvastatin and amlodipine, and atorvastatin and aspirin combinations (Figure 1).

**Figure 2:**
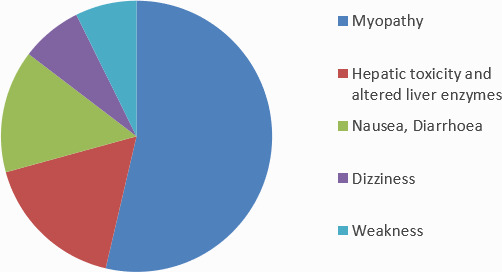
The most commonly observed side effects with the statins.

Among 45 respondents, 4 (8.8%) reported that they have not observed any side effects in their patients till now. Myopathy was reported by 22 (48.8%) respondents as the most common side effects in their patients (Figure 2).

## DISCUSSION

In this study, the incidence of dyslipidemia was reported to be greater in male than in the female population. A similar finding was seen in the GENOA study done by John 2004 for the Mayo clinic and foundation.^6^ The statins 3-hydroxymethylglutaryl coenzyme A (HMG- CoA) reductase inhibitors represent drugs of the first choice for the treatment of hypercholesterolemia.^4^ In this study, the statins with a score of 4.25 were accounted as the most preferred by the doctors, among the different group of drugs effective for treating dyslipidemia. The clinical effects of statins direct toward potential therapeutic equivalence; although they differ in other important aspects such as pharmacokinetic properties.^7^

The selection of a particular drug should be primarily based on clinical outcome data. However, costs and in certain situations the pharmacokinetic profile including the interaction potential of the Statins should be taken into account. Agents inhibiting cytochrome P450 3A4 (CYP3A4) should be discouraged if a patient is on atorvastatin, lovastatin, or simvastatin.^4^ Drugs or food that inhibit CYP3A4 can markedly increase the plasma levels of simvastatin and lovastatin given their low bioavailability, and to a less extent, that of atorvastatin, with a consequent increase in the risk of rhabdomyolysis and muscular toxicity.^10^

The pharmacokinetics and interaction potentials of the possible over the counter (OTC) candidate Statins simvastatin, lovastatin, fluvastatin, and pravastatin are different. Simvastatin and lovastatin are mainly metabolized by cytochrome P450 3A4, fluvastatin is metabolized by CYP2C9, and pravastatin is excreted largely unchanged. On the pharmacokinetic basis, fluvastatin and pravastatin can be better choices than simvastatin or lovastatin as OTC statin.^11^

In this study, Atorvastatin was found to be the most preferred type of drug followed by rosuvastatin and simvastatin as shown in Table 2. Although all the statins type shows similar efficacy in treating dyslipidemia, there was propounding selectivity towards atorvastatin. In this study, the dominating score of 4.48 atorvastatin was reported to be the most selected type of statin. Release of this drug in 1997, which coincided with the United States Food and Drug Administration's approval of Direct-to-Consumer Pharmaceutical Advertising, allowed for the broadcast advertisement of prescription drugs. These events made atorvastatin the main drug of choice, even though there is no strong evidence supporting large differences among statins in human or animal studies.^8,9^ Based on an approximately 30% reduction of low-density lipoprotein (LDL), atorvastatin (5 mg/day), and simvastatin (10 mg/day) are the most potent agents whereas 40 mg of lovastatin or pravastatin and 60 mg of fluvastatin are needed to reach this therapeutic target.^4^ These impacts might have influenced the doctors in this study also, to incline towards the preference of atorvastatin to such a high extent.

About 60% of the respondent doctors did not prefer the fixed-dose combinations of the statins. Among the different combinations available in the market, the combination of atorvastatin and ezetimibe (17.7%) was found to be the most preferred one. All common lipid-lowering drugs like fibrates, nicotinic acid derivatives, and statins, have been associated with myopathy. More and more so far unknown subtypes of myopathy are identified. Hence to avoid the combinations are usually recommended. The most common side effects, the doctors encountered in their patients was different forms of myopathy. It accounted to be experienced by 22 (48.8%) of the doctors in their patients as the main side effect. In the large clinical trials with various statins, the rate of muscular side effects consistently has been reported to the range at around 5%.^12^

Especially the high doses and combination with Fibrates was responsible for the life-threatening consequence.^12^ Patients with multiple medical co-morbidities are at increased risk of adverse effects from long-term statins use. Myalgia is the most common side effect of Statin use, with documented rates from 1-10%. Rhabdomyolysis is the most serious adverse effect from statin use, though it occurs quite rarely (less than 0.1%).^13^

The limitation of the study is that it was conducted in a small population and based on the practicing doctors from one hospital only, so the finding from this study cannot be generalized for the whole population.

## CONCLUSIONS

This descriptive study shows that statins are the main group of drugs doctors preferred for the treatment of dyslipidemia. And among different statin which is assumed to have similar efficacy, atorvastatin was the most favored one. Most of the doctors were not in favor of any kind of fixed-dose combination of statins. Among the doctors who prescribe the combination, the combination of atorvastatin and ezetimibe was most popular. The most common side effects with satin were myopathy, according to the majority of prescribing doctors.

For more conclusive and convincing results a multi-centric study with a sufficient sample size has to be taken. The research modalities should be elaborated more.
